# Maternal Concentrations of Persistent Organochlorine Pollutants and the Risk of Asthma in Offspring: Results from a Prospective Cohort with 20 Years of Follow-up

**DOI:** 10.1289/ehp.1206397

**Published:** 2013-10-25

**Authors:** Susanne Hansen, Marin Strøm, Sjurdur F. Olsen, Ekaterina Maslova, Panu Rantakokko, Hannu Kiviranta, Dorte Rytter, Bodil H. Bech, Linda V. Hansen, Thorhallur I. Halldorsson

**Affiliations:** 1 Centre for Fetal Programming, Department of Epidemiology Research, Statens Serum Institut, Copenhagen, Denmark; 2 Department of Nutrition, Harvard School of Public Health, Boston, Massachusetts, USA; 3 Department of Environmental Health, National Institute for Health and Welfare (THL), Kuopio, Finland; 4 Department of Public Health, Section for Epidemiology, Aarhus University, Denmark; 5 Faculty of Food Science and Nutrition, University of Iceland, Reykjavik, Iceland; 6 Unit for Nutrition Research, Landspitali University Hospital, Reykjavik, Iceland

## Abstract

Background: Previous findings suggest that developmental exposures to persistent organochlorine pollutants (POPs) may be detrimental for the development of the immune system in the offspring. Whether these suspected immunoregulatory effects persist beyond early childhood remains unclear.

Objectives: The objective of this study was to evaluate the association between maternal serum concentrations of POPs and the risk of asthma in offspring after 20 years of follow-up.

Methods: A birth cohort with 965 women was formed in 1988–1989 in Aarhus, Denmark. Concentrations of six polychlorinated biphenyls (PCBs) (congeners 118, 138, 153, 156, 170, 180), hexachlorobenzene (HCB), and dichlorodiphenyldichloroethylene (*p,p*´-DDE) were quantified in maternal serum (*n* = 872) collected in gestation week 30. Information about offspring use of asthma medications was obtained from the Danish Registry of Medicinal Product Statistics.

Results: Maternal serum concentrations of HCB and dioxin-like PCB-118 were positively associated with offspring asthma medication use after 20 years of follow-up (*p* for trend < 0.05). Compared with subjects in the first tertile of maternal concentration, those in the third tertile of PCB-118 had an adjusted hazard ratio (HR) of 1.90 (95% CI: 1.12, 3.23). For HCB the HR for the third versus the first tertile of maternal concentration was 1.92 (95% CI: 1.15, 3.21). Weak positive associations were also estimated for PCB-156 and the non-dioxin-like PCBs (PCBs 138, 153, 170, 180). No associations were found for *p,p*´-DDE.

Conclusions: Maternal concentrations of PCB-118 and HCB were associated with increased risk of asthma in offspring followed through 20 years of age.

Citation: Hansen S, Strøm M, Olsen SF, Maslova E, Rantakokko P, Kiviranta H, Rytter D, Bech BH, Hansen LV, Halldorsson TI. 2014. Maternal concentrations of persistent organochlorine pollutants and the risk of asthma in offspring: results from a prospective cohort with 20 years of follow-up. Environ Health Perspect 122:93–99; http://dx.doi.org/10.1289/ehp.1206397

## Introduction

Polychlorinated biphenyls (PCBs) are a group of structurally related organic compounds that, because of their inertness and thermal stability, have been extensively used in various industrial and commercial applications. Although manufacturing of PCBs was banned in the late 1970s due to concerns of possible adverse effects in humans, these compounds still persist in the environment (Dyke et al. 2003). Similar concerns in the 1970s also led to ban or severe restrictions on the use of the organochlorine pesticides hexachlorobenzene (HCB) and dichlorodiphenyltrichloroethane (*p,p*´-DDT). Because of their lipophilic and persistent nature, *p,p*´-DDT and its metabolite dichlorodiphenyldichloroethylene (*p,p*´-DDE), HCB, and PCBs accumulate through the food chain, with seafood currently being the main route of human exposure (Halldorsson et al. 2007; Thompson and Boekelheide 2013). On entering the human body, these compounds dissolve in lipids but they can also bind to proteins in blood (Mohammed et al. 1990), and during pregnancy they are readily transported across the placenta (Covaci et al. 2002; Park et al. 2008).

There is some evidence to suggest that prenatal exposure to PCBs may adversely affect development and maturation of the immune system (Lundqvist et al. 2006). Studies focusing on background prenatal exposures to PCBs have reported associations with increased respiratory and otitis media infections (Dallaire et al. 2006; Glynn et al. 2008; Weisglas-Kuperus et al. 2000), altered immune cell counts (Glynn et al. 2008; Jusko et al. 2011; Weisglas-Kuperus et al. 2000), and reduced antibody responses to childhood vaccines (Heilmann et al. 2006; Weisglas-Kuperus et al. 2000). Reports on atopic diseases such as asthma and wheeze have been divergent, with two studies reporting positive associations (Grandjean et al. 2010; Stolevik et al. 2011) and another reporting an inverse association (Weisglas-Kuperus et al. 2000, 2004). At least two studies have reported positive associations between prenatal exposures to *p,p*´-DDE and asthma in offspring at 4–9 years of age (Karmaus et al. 2001; Sunyer et al. 2005), whereas evidence for immunological effects of HCB has mostly been obtained from animal studies (Ezendam et al. 2005; Michielsen et al. 1999).

Divergent findings on associations between prenatal exposures to these persistent organochlorine pollutants (POPs) and asthma may relate to the fact that most studies have not had follow-up beyond 6–7 years of age, which would facilitate more accurate diagnoses of permanent asthma when coughing and wheeze symptoms have stabilized. Divergent findings may also relate to differences in relative and absolute concentrations of different organochlorine compounds across studies due to temporal and regional differences (Longnecker et al. 2003). Furthermore, previous studies have mostly relied on self- or parental report to assess asthma, which may be prone to misclassification (Peat et al. 1992, 2001).

The aim of this study was to investigate the association between maternal serum concentrations of PCBs, HCB, and *p,p*´-DDE in a cohort of environmentally exposed Danish pregnant women and risk of asthma in offspring after 20 years of follow-up.

## Methods

*Study population*. In Aarhus, Denmark, the Danish Fetal Origins 1988–1989 Cohort was formed and included 965 (80%) of 1,212 eligible women with singleton pregnancies who attended a large prenatal clinic during the study period. The cohort has been described in detail elsewhere (Olsen et al. 1995). Briefly, the data collection included a self-administered dietary questionnaire that was sent to the women 1 week before their scheduled 30th week antenatal visit. During the midwife visit the women participated in a face-to-face interview and gave information about diet, lifestyle, anthropometry, medical history, and socioeconomic status. A blood sample was collected and immediately separated into erythrocytes, serum, and plasma, and was frozen at –20 °C. Information about the health of the mothers and birth outcomes was collected from the Danish Medical Birth Registry (Copenhagen, Denmark).

*Follow-up of children*. In 2008 a follow-up study was conducted including the offspring whose mothers were originally enrolled. Health information was obtained from the Danish population-based disease registries, and the offspring were also contacted and asked to complete a self-administered web-based questionnaire covering anthropometry, lifestyle, diet, and health. The questionnaire included standardized questions on asthma from the International Study of Asthma and Allergies in Childhood (ISAAC) (Asher et al. 1995).

The study was approved by the Danish Data Protection Agency and the Danish Council of Ethics (Reference No. 20070157), and all participants gave written consent before inclusion in the study.

*Exposure measures*. A total of six PCBs (congeners 118, 138, 153, 156, 170, and 180), *p,p*´-DDE, and HCB were measured in 200-μL aliquots from archived maternal serum using an extension of previously published methods (Rantakokko et al. 2009) described in more detail elsewhere (Bjermo et al. 2013). In each batch of samples (*n* = 25), two blanks were included to control for possible laboratory contamination, in addition to two positive control samples. Average recoveries of measured POPs in the control samples were 97–106% of the certified values. The between-assay coefficient of variation was 4.0% (at 0.17 ng/mL) for PCB-118; 2.7% (at 0.94 ng/mL) for PCB-153; 4.0% (at 0.08 ng/mL) for HCB; and 2.1% (at 11.5 ng/mL) for *p,p*´-DDE. The limits of quantification (LOQs) for the PCBs and *p,p*´-DDE were 2–5 pg/mL. The LOQ for HCB was 25 pg/mL. The concentrations of the six PCB congeners, *p,p*´-DDE, and HCB were above the LOQs in all samples.

*Outcome measures*. Participants were classified as asthma cases using a previously validated definition based on medication data (Moth et al. 2007). We used Danish personal identification numbers to link each participant to the Registry of Medicinal Product Statistics (RMPS; Copenhagen, Denmark), which has collected information about all prescriptions redeemed in Danish pharmacies since 1995, and extracted information about all medications used to treat asthma [Anatomical Therapeutic Chemical Classification System (ATC; World Health Organization, Oslo, Norway) codes R03A, R03B, R03C, and R03D].

Participants who were prescribed medication(s) used for the treatment of asthma from 6 to 20 years of age were classified as cases, except for those who received prescriptions for liquid β2-agonists only, or who received only one prescription for an inhaled β2-agonist or an inhaled steroid (Moth et al. 2007). The date when an offspring became an asthma case for the first time was used in the analyses. As secondary outcome measures, we used three additional asthma definitions. Self-reported lifetime doctor diagnosis of asthma was assessed from a standardized ISAAC question on the follow-up questionnaire at 20 years of age and defined as offspring with a positive answer to this question. Offspring with a self-reported doctor diagnosis were furthermore asked if they had used any asthma medications in the previous 12 months, to define self-reported current use of asthma medications. We also used hospital diagnoses for asthma from the mandatory Danish National Patient Register (DNPR; Copenhagen, Denmark), which contains information about all hospital admissions in Denmark since 1977 and emergency department and outpatient contacts since 1995. We defined a hospital asthma case as offspring with any asthma diagnosis (including hospital in-, out-, and emergency department patients) by the *International Classification of Diseases, 10th Revision* (ICD-10; National Board of Health, Denmark 1994) codes J45.0, J45.1, J45.2, J45.8, J45.9 J46.9, and *8th Revision* (ICD-8; National Board of Health, Denmark 1977) codes 493.00, 493.01, 493.02, 493.08, 493.09) from birth to 20 years of age. We used the date of the first hospital diagnosis in the analyses.

*Mother–child pairs available for analysis*. For the 965 women originally enrolled in the study, a sufficient amount of serum from week 30 of gestation was available for 872 subjects (90%). These women and their offspring form the basis for this study. There were no major differences in baseline characteristics between participants and nonparticipants (*n* = 93) (data not shown). Information on use of medication (from 1995, offspring ≈ 6 years of age) and hospital diagnosis (from birth) were available for all offspring. Self-reported lifetime diagnosis of asthma and self-reported medication use in the preceding 12 months were available for 654 offspring who filled out the web-based questionnaire at the 2008 follow-up.

*Statistical analyses*. We used the median and 10th and 90th percentiles to describe skewed variables, the mean ± SD for normal variables, and percentages for categorical variables. The dioxin-like PCBs (congeners 118 and 156) and the non-dioxin-like PCBs (congeners 138, 153, 170, and 180) and the sum of all six congeners were analyzed separately and aggregated on the bases of molar concentrations. Otherwise, wet-weight concentrations (nanograms per milliliter) were used for individual PCB congeners, HCB, and *p,p*´-DDE. In our primary analysis we examined the association between maternal PCB, HCB, and *p,p*´-DDE serum concentrations and offspring risk of asthma (based on asthma medication use) during 20 years of follow-up using Cox regression models. Maternal concentrations of the organochlorine compounds were divided into tertiles, and hazard ratios (HRs) and 95% CIs were calculated using age as the underlying time scale. Participants were thus considered at risk of asthma from their age at the start of follow-up in 1995, until time of becoming an asthma case (based on asthma use) or the defined end of follow-up (end of 2008), whichever came first. None of the participants were censored for other reasons, such as death or emigration. Using this model we assumed that all offspring were free of asthma at the start of follow-up in 1995, when the offspring were around 6 years of age. Any prescription of asthma medication before 1995 was assumed to be related to coughing and wheezing rather than asthma (Henderson et al. 2008). Visual inspection of cumulative residual plots did not indicate violations to the assumption of proportional hazards (data not shown). When examining associations between maternal concentrations of POPs and offspring asthma, we performed trend tests by assigning the median concentration to each exposure level (tertile) and included this in the regression models as a continuous variable.

To test the stability of our findings, both with respect to the asthma definition and the assumption that offspring were disease free until 6 years of age, we also estimated associations of PCB, HCB, and *p,p*´-DDE with first asthma diagnosis in the DNPR from birth to 20 years of age using Cox regression. Furthermore, we examined the relation between PCB, HCB, and *p,p*´-DDE, self-reported lifetime diagnosis of asthma, and self-reported current use of asthma medication using logistic regression.

Information on covariates was obtained from questionnaires completed by the mothers during pregnancy and from maternal birth records, and the following covariates were included as adjustment factors in the multivariate models: maternal age (continuous), parity (0, 1, ≥ 2), prepregnancy body mass index (BMI; kilograms per meter squared) (< 18.5, 18.5 to < 25, 25 to < 30, ≥ 30), maternal education (elementary school, high school or technical school, university education, higher academic education, other education, or missing), maternal smoking (nonsmokers, or < 5, > 5 to 15, > 15 cigarettes/day), maternal concentrations of cholesterol and triglycerides (millimoles per liter), child’s birth weight (continuous), and child’s sex. We considered gestational age to be a potential intermediate variable and therefore excluded it from the model. Information on maternal smoking and education was missing for 6% of participants, and 3.5% had missing information on maternal prepregnancy BMI. Complete information was available for the remaining covariates. Missing covariate values were substituted using multiple imputation (PROC MI) in SAS. We did not adjust for fish intake to avoid overadjusting our model (Halldorsson et al. 2008).

All tests were two-sided and statistical significance was considered at *p* < 0.05. All analyses were carried out using SAS statistical software (version 9.3; SAS Institute Inc., Cary, NC, USA).

## Results

Characteristics of the study population are shown in Table 1. The median maternal serum concentration from gestational week 30 was 1.37 ng/mL for PCB-153, 0.54 ng/mL for HCB, and 2.47 ng/mL for *p,p*´-DDE. Approximately 13% of the offspring had been prescribed asthma medications, whereas 14% had a self-reported lifetime diagnosis of asthma. The proportion of offspring with a hospital asthma diagnosis was 4%, whereas 7% had self-reported use of asthma medication in the previous 12 months (Table 1).

**Table 1:** Maternal and offspring characteristics of 872 mother–child pairs.

Characteristic	Median (10th, 90th percentiles), mean ± SD, or *n* (%)
Maternal	
Serum concentration (ng/mL)	
PCB-118	0.17 (0.09, 0.30)
PCB-138	0.74 (0.41, 1.25)
PCB-153	1.37 (0.76, 2.26)
PCB-156	0.10 (0.06, 0.16)
PCB-170	0.37 (0.21, 0.59)
PCB-180	0.67 (0.38, 1.10)
Sum of PCBs (pmol/mL)	9.23 (5.32, 15.11)
HCB	0.54 (0.31, 0.87)
*p,p*´-DDE	2.47 (1.03, 5.66)
Maternal age at birth (years)	29.0 ± 4.2
Fish intake (g/day)	19.3 ± 15.6
Alcohol (g/day)	2.9 ± 3.8
Maternal cholesterol (mmol/L)	7.3 ± 1.3
Maternal triglycerides (mmol/L)	2.4 ± 0.8
Previous births	
0	500 (57.3)
1	279 (32.0)
≥ 2	93 (10.7)
Cigarettes/day during pregnancy	
0	493 (56.5)
> 0–5	99 (11.4)
> 5 to 15	188 (21.6)
> 15	41 (4.7)
Missing	51 (5.9)
Maternal education	
Elementary school	103 (11.8)
High school or technical school	207 (23.7)
University	296 (33.9)
Higher academic	125 (14.3)
Other education	89 (10.2)
Missing	52 (6.0)
Prepregnancy BMI (kg/m^2^)	
< 18.5	89 (10.2)
18.5 to < 25	676 (77.5)
25 to < 30	53 (6.1)
> 30	23 (2.6)
Missing	31 (3.6)
Offspring	
Registry-based diagnoses (*n *= 872)	
Asthma medication	111 (12.7)
Asthma hospitalization	32 (3.7)
Self-reported diagnoses (*n *= 654)	
Lifetime doctor diagnosis	89 (13.6)
Current asthma medication use	48 (7.3)
Birth weight (g)	3493.7 ± 579.9
Gestational age (days)	282.3 ± 11.8
Boys	461 (52.9)
Age at follow-up (years)	19.6 ± 0.49

Of those who reported ever being diagnosed with asthma by a doctor, 72% were also classified as cases based on the medication registry data; 76% of those classified as cases based on the medication registry data self-reported a diagnosis. Only 4% of those who were not classified as cases based on the medication registry data had a self-reported diagnosis, and only 3.5% of those who did not report a diagnosis were classified as cases based on the medication registry data. The agreement between the hospital diagnoses and the other outcomes was lower both for cases and noncases (20–31%) (data not shown).

Compared with mothers who had PCB concentrations in the first and second tertiles, mothers in the third tertile of PCB concentration were older (30.4 years of age vs. 27.6 and 29.1 in the first tertile and second tertile, respectively), had higher mean fish intake (20.6 g/day vs. 18.8 and 18.4), higher alcohol intake (3.4 g/day vs. 2.5 and 2.9), were more likely to be nulliparous (64.6% vs. 52.4 and 55.0), and had higher academic education (16.8% vs. 13.6 and 16.4) (Table 2). Similar associations were also observed across the tertiles of HCB and *p,p*´-DDE exposures. There was no difference in maternal prepregnancy BMI across PCB tertiles. However, for HCB and *p,p*´-DDE maternal prepregnancy BMI was higher in the third tertiles compared with the first tertile (22.1 kg/m^2^ vs. 21.0 and 21.8 vs. 20.9 for HCB and *p,p*´-DDE, respectively). Offspring of mothers in the third tertiles of PCB and HCB exposure had slightly shorter gestation (281 days) compared with offspring of mothers in the first (283 days) and second tertile (283 days). This difference was not observed across the *p,p*´-DDE tertiles. There was no difference in birth weight across any of the exposure tertiles. More boys than girls were born to mothers in the third tertile of PCB exposure (58.1% vs. 49.0% in the first tertile). For all four outcomes of asthma, there were higher numbers of asthma cases in the second and third tertiles of maternal PCB and HCB exposures compared to the first tertile.

**Table 2:** Maternal and offspring characteristics of 872 mother–child pairs across tertiles of maternal PCB, HCB, and *p,p´*-DDE serum concentrations.

Characteristic	1st tertile	2nd tertile	3rd tertile	*p*-Value^*a*^
PCB concentration (pmol/mL) (range)	1.4–7.7	7.7–11.1	11.1–80.6
Maternal
Maternal age at birth (years)	27.6 ± 4.1	29.1 ± 3.9	30.4 ± 4.2	< 0.001
Fish intake (g/day)	18.8 ± 16.3	18.4 ± 13.4	20.6 ± 16.7	0.21
Alcohol (g/day)	2.5 ± 3.7	2.9 ± 3.3	3.4 ± 4.2	0.01
Previous births (% nulliparous)	152 (52.4)	160 (55.0)	188 (64.6)	0.02
Smoking during pregnancy (% no)	157 (57.5)	182 (66.4)	154 (56.2)	0.09
Maternal education (% higher academic)	37 (13.6)	42 (15.4)	46 (16.8)	0.02
Prepregnancy BMI (kg/m^2^)	21.5 ± 3.0	21.5 ± 3.1	21.3 ± 3.2	0.72
Offspring
Birth weight (g)	3514.8 ± 507.7	3503.3 ± 563.8	3462.7 ± 541.1	0.47
Gestational age (days)	283 ± 10.8	283 ± 12.2	281 ± 12.3	0.03
Sex (% boys)	142 (49.0)	150 (51.6)	169 (58.1)	0.08
Asthma medication (*n* cases)	33	36	42	0.53
Asthma hospital diagnoses (*n* cases)	8	12	12	0.60
Self-reported diagnoses (*n* cases)	22	34	33	0.26
Current self-reported medication use (*n* cases)	11	18	19	0.35
HCB concentrations (ng/mL) (range)	0.1–0.5	0.5–0.6	0.6–2.5
Maternal
Maternal age at birth (years)	28.0 ± 4.1	28.9 ± 3.8	30.2 ± 4.2	< 0.001
Fish intake (g/day)	18.1 ± 14.5	19.5 ± 15.7	20.2 ± 16.3	0.24
Alcohol (g/day)	2.6 ± 3.8	3.0 ± 3.5	3.2 ± 3.9	0.14
Previous births (% nulliparous)	140 (48.3)	168 (57.7)	192 (66.0)	< 0.001
Smoking during pregnancy (% no)	162 (59.6)	162 (59.3)	169 (61.2)	0.64
Maternal education (% higher academic)	36 (13.2)	44 (16.2)	45 (16.3)	0.22
Prepregnancy BMI (kg/m^2^)	21.0 ± 2.5	21.2 ± 2.4	22.1 ± 4.0	< 0.001
Offspring
Birth weight (g)	3483.2 ± 503.1	3534.7 ± 534.8	3463.0 ± 572.8	0.25
Gestational age (days)	283 ± 11.7	283 ± 10.9	281 ± 12.8	0.04
Sex (% boys)	147 (50.7)	155 (53.3)	159 (54.6)	0.63
Asthma medication (*n* cases)	28	35	48	0.04
Asthma hospital diagnoses (*n* cases)	8	12	12	0.60
Self-reported diagnoses (*n* cases)	20	34	35	0.07
Current self-reported medication use (*n* cases)	6	20	22	0.01
*p,p*´-DDE concentrations (ng/mL) (range)	0.2–1.9	1.9–3.2	3.3–38.8
Maternal
Maternal age at birth (years)	28.0 ± 4.2	28.8 ± 4.0	30.3 ± 4.1	< 0.001
Fish intake (g/day)	19.1 ± 14.7	17.7 ± 14.4	20.9 ± 17.3	0.05
Alcohol (g/day)	2.4 ± 3.2	3.1 ± 3.9	3.4 ± 4.1	0.01
Previous births (% nulliparous)	148 (51.0)	171 (58.8)	181 (62.2)	0.05
Smoking during pregnancy (% no)	162 (58.5)	174 (64.4)	157 (57.3)	0.59
Maternal education (% higher academic)	35 (12.6)	38 (14.1)	52 (19.0)	0.03
Prepregnancy BMI (kg/m^2^)	20.9 ± 2.7	21.5 ± 2.9	21.8 ± 3.6	0.003
Offspring
Birth weight (g)	3499.9 ± 533.5	3494.7 ± 519.8	3486.6 ± 561.1	0.96
Gestational age (days)	283 ± 11.7	282 ± 11.8	282 ± 12.0	0.49
Sex (% boys)	146 (50.3)	159 (54.6)	156 (53.6)	0.56
Asthma medication (*n* cases)	37	34	40	0.76
Asthma hospital diagnoses (*n* cases)	10	11	11	0.97
Self-reported diagnoses (*n* cases)	30	31	28	0.89
Current self-reported medication use (*n* cases)	16	16	16	0.99
Values are mean ± SD, *n*, or *n* (%). ^***a***^Differences in maternal age, fish intake, alcohol, prepregnancy BMI, birth weight, gestational age across tertiles of maternal POP concentrations were evaluated using *F*-test. Differences in previous births, smoking, maternal education, sex, asthma medication, asthma hospital diagnoses, and self-reported diagnoses, self-reported medication across tertiles of maternal POP concentrations were evaluated using chi-square test.				

HCB exposure in the third tertile was significantly associated with asthma classified according to medication use in both unadjusted (HR = 1.78; 95% CI: 1.12, 2.84) and adjusted (HR = 1.92; 95% CI: 1.15, 3.21) models (Table 3). We found that for the dioxin-like PCB-118 and PCB-156 combined, HRs of asthma medication use increased across maternal tertiles of exposure. The association was strongest for maternal concentrations of PCB-118 with offspring asthma medication use after adjustment for covariates (third vs. first tertile 1.90; 95% CI: 1.12–3.23). Although there were no significant associations between maternal concentrations of the sum of all PCBs and the non-dioxin-like PCBs with asthma medication use, HRs were positive and increased with increasing tertiles of maternal exposure. There was no association between maternal concentrations of *p,p*´-DDE and offspring asthma medication. Overall, additional adjustment for gestational age did not alter any of the results (data not shown).

**Table 3:** Associations between maternal PCBs, HCB, and *p,p´*-DDE serum concentrations and offspring asthma medication use after 20 years of follow-up (*n* = 872) [HR^*a*^ (95% CI)].

POPs in tertiles (range)	Raw model	Adjusted model^*b*^
PCB–118 (ng/mL)
1st (0.02–0.14)	1.00	1.00
2nd (> 0.14–0.20)	1.50 (0.92, 2.45)	1.60 (0.96, 2.66)
3rd (> 0.20–0.61)	1.69 (1.05, 2.73)	1.90 (1.12, 3.23)
*p* for trend^*c*^	0.04	0.02
PCB–156 (ng/mL)
1st (0.01–0.08)	1.00	1.00
2nd (> 0.08–0.12)	1.33 (0.82, 2.15)	1.38 (0.84, 2.26)
3rd (> 0.12–0.92)	1.43 (0.90, 2.29)	1.45 (0.85, 2.46)
*p* for trend^*c*^	0.15	0.20
Dioxin-like PCBs (pmol/mL)^*d*^
1st (0.13–0.67)	1.00	1.00
2nd (> 0.67–0.96)	1.45 (0.90, 2.35)	1.56 (0.95, 2.56)
3rd (> 0.96–4.10)	1.59 (0.99, 2.55)	1.75 (1.02, 2.98)
*p* for trend^*c*^	0.07	0.05
Non-dioxin-like PCBs (pmol/mL)^*e*^
1st (1.23–7.09)	1.00	1.00
2nd (> 7.10–10.12)	1.10 (0.69, 1.77)	1.15 (0.70, 1.86)
3rd (> 10.12–76.55)	1.30 (0.83, 2.06)	1.30 (0.78, 2.17)
*p* for trend^*c*^	0.24	0.32
Sum of all PCBs (pmol/mL)^*f*^
1st (1.43–7.72)	1.00	1.00
2nd (> 7.73–11.11)	1.10 (0.68, 1.76)	1.15 (0.70, 1.87)
3rd (> 11.11–80.65)	1.30 (0.83, 2.06)	1.30 (0.78, 2.17)
*p* for trend^*c*^	0.24	0.32
HCB (ng/mL)
1st (0.07–0.45)	1.00	1.00
2nd (> 0.45–0.63)	1.26 (0.77, 2.07)	1.31 (0.79, 2.17)
3rd (> 0.63–2.45)	1.78 (1.12, 2.84)	1.92 (1.15, 3.21)
*p* for trend^*c*^	0.01	0.01
*p,p*´–DDE (ng/mL)
1st (0.20–1.86)	1.00	1.00
2nd (> 1.86–3.24)	0.91 (0.57, 1.44)	0.92 (0.5, 1.47)
3rd (> 3.25–38.77)	1.09 (0.70, 1.71)	1.09 (0.67, 1.77)
*p* for trend^*c*^	0.62	0.64
^***a***^Estimated in a Cox regression model with age as the underlying time scale. ­^***b***^Adjusted for maternal age, prepregnancy BMI, parity, maternal smoking, maternal education, maternal alcohol intake, maternal cholesterol, maternal triglycerides, child birth weight, and sex. ^***c***^Estimated with median concentrations in each tertile entered in the Cox regression model as a continuous variable. ^***d***^Sum of PCB congeners 118 and 156. ^***e***^Sum of PCB congeners 138, 153, 170, and 180. ^***f***^Sum of PCB congeners 118, 138, 153, 156, 170, 180.		

In most cases, maternal concentrations of PCBs and HCB were positively associated with asthma hospital diagnoses, self-reported lifetime diagnoses, and self-reported current medication use (Table 4). The strongest associations were found between maternal concentrations and PCB-118 and HCB and self-reported current use of asthma medication. No association was observed between maternal concentrations of *p,p*´-DDE and any asthma outcomes.

**Table 4:** Associations between maternal PCBs, HCB, and *p,p´*-DDE serum concentrations and the risk of offspring hospital diagnoses (*n* = 872), self-reported doctor diagnosis of asthma (*n* = 654), and self-reported current medication use (*n* = 654) in the adjusted model^*a*^.

POPs in tertiles (range)	Asthma hospital diagnoses [HR^*b*^ (95% CI) *n *= 872]	Self-reported lifetime diagnosis [Odds ratio^*c*^ (95% CI) *n *= 654]	Current self-reported medication use [Odds ratio^*c*^ (95% CI) *n *= 654]
PCB-118 (ng/mL)
1st (0.02–0.14)	1.00	1.00	1.00
2nd (> 0.14–0.20)	0.85 (0.50, 1.46)	1.76 (0.94, 3.26)	1.90 (0.80, 4.52)
3rd (> 0.20–0.61)	1.31 (0.78, 2.20)	1.73 (0.90, 3.31)	2.49 (1.03, 5.99)
*p* for trend^*d*^	0.32	0.16	0.05
PCB-156 (ng/mL)
1st (0.01–0.08)	1.00	1.00	1.00
2nd (> 0.08–0.12)	1.38 (0.85, 2.25)	1.52 (0.83, 2.80)	1.75 (0.75, 4.07)
3rd (> 0.12–0.92)	0.94 (0.53, 1.66)	1.41 (0.73, 2.71)	2.01 (0.82, 4.93)
*p* for trend^*d*^	0.76	0.38	0.15
Dioxin-like PCBs (pmol/mL)^*e*^
1st (0.13–0.67)	1.00	1.00	1.00
2nd (> 0.67–0.96)	1.36 (0.84, 2.21)	1.83 (1.00, 3.36)	1.80 (0.79, 4.11)
3rd (> 0.96–4.10)	1.00 (0.57, 1.75)	1.45 (0.75, 2.83)	1.90 (0.79, 4.59)
*p* for trend^*d*^	0.40	0.40	0.19
Non-dioxin-like PCBs (pmol/mL)^*f*^
1st (1.23–7.09)	1.00	1.00	1.00
2nd (> 7.10–10.12)	1.01 (0.61, 1.68)	1.40 (0.77, 2.56)	1.35 (0.60, 3.00)
3rd (> 10.12–76.55)	1.19 (0.69, 2.04)	1.27 (0.67, 2.44)	1.48 (0.63, 3.46)
*p* for trend^*d*^	0.40	0.57	0.39
Sum of all PCBs (pmol/mL)^*g*^
1st (1.43–7.72)	1.00	1.00	1.00
2nd (> 7.73–11.11)	1.12 (0.68, 1.84)	1.59 (0.87, 2.90)	1.59 (0.71, 3.60)
3rd (> 11.11–80.65)	1.22 (0.71, 2.09)	1.30 (0.67, 2.51)	1.63 (0.68, 3.88)
*p* for trend^*d*^	0.30	0.59	0.32
HCB (ng/mL)
1st (0.07–0.45)	1.00	1.00	1.00
2nd (> 0.45–0.63)	1.66 (0.66, 4.13)	1.79 (0.97, 3.31)	3.38 (1.29, 8.85)
3rd (> 0.63–2.45)	1.84 (0.70, 4.86)	1.85 (0.97, 3.51)	4.18 (1.57, 11.15)
*p* for trend^*d*^	0.24	0.09	0.01
*p,p*´–DDE (ng/mL)
1st (0.20–1.86)	1.00	1.00	1.00
2nd (> 1.86–3.24)	1.17 (0.49, 2.80)	0.96 (0.55, 1.69)	0.95 (0.45, 1.98)
3rd (> 3.24–38.77)	1.14 (0.46, 2.85)	0.80 (0.44, 1.45)	0.86 (0.40, 1.85)
*p* for trend^*d*^	0.80	0.48	0.91
^***a***^Adjusted for maternal age, prepregnancy BMI, parity, maternal smoking, maternal education, maternal alcohol intake, maternal cholesterol, maternal triglycerides, child birth weight, and sex. ^***b***^Estimated in a Cox regression model with age as the underlying time scale. ^***c***^Estimated in a logistic regression model. ^***d***^Estimated with median concentrations in each tertile entered in the Cox or logistic regression models as a continuous variable. ^***e***^Sum of PCB congeners 118 and 156. ^***f***^Sum of PCB congeners 138, 153, 170, and 180. ^***g***^Sum of PCB congeners 118, 138, 153, 156, 170, 180.			

The correlations between the compounds were high (Spearman *r* = 0.53–0.99), which made mutual adjustments problematic. However, when the analyses with PCB-118 and HCB (Spearman *r* = 0.77) were mutually adjusted for each other in addition to the other covariates, we found weakened associations and wider CIs for PCB-118 (HR = 1.47; 95% CI: 0.75, 2.86) and for HCB (HR = 1.58; 95% CI: 0.83, 3.01) with offspring asthma medication use comparing the third tertile to the first tertile of exposure (data not shown).

## Discussion

In this study with 20 years of follow-up we observed positive associations between maternal serum concentrations of PCBs and HCB and offspring use of asthma medications. For the PCBs, the strongest associations were found for the dioxin-like PCB-118. Similar associations were observed when we used three additional asthma outcomes, including hospital diagnoses and self-reported diagnoses. No associations were found for maternal concentrations of *p,p*´-DDE and offspring asthma.

To our knowledge, only a few studies have examined developmental exposures to PCBs in relation to wheezing and asthma, and their results have varied, with some reporting positive associations (Grandjean et al. 2010; Stolevik et al. 2011), whereas another reported an inverse association (Weisglas-Kuperus et al. 2000, 2004). In contrast with these studies, we also estimated associations with specific PCB congeners, in addition to the sum of all quantified PCBs. Of the six quantified PCB congeners, only dioxin-like PCB-118 was significantly associated with offspring asthma, and a stronger association was observed for the two dioxin-like congeners combined (PCBs 118 and 156) compared with the non-dioxin-like congeners (PCBs 138, 153, 170, and 180) and the sum of all PCBs.

The suspected influence of the dioxin-like PCBs on immunoregulation is, at least partly, thought to be related to interactions with the aryl hydrocarbon receptor (AhR). AhR-mediated responses with dioxin and dioxin-like compounds are thought to contribute to asthma pathogenesis through increased expression of inflammatory cytokines, including tumor necrosis factor-α and interleukin-1β, which in turn can induce mucin production, cell chemotaxis, and immunoglobulin E (IgE) production (Chiba et al. 2012). Whether AhR-mediated responses are relevant to effects of developmental exposures to dioxin-like PCBs is unclear. Stronger associations of PCB-118 with asthma in our study are also indirectly supported by two studies. PCB-118 was positively associated with cord IgE concentrations in a cross-sectional study of children from the Slovak Republic, but no associations were reported for the other quantified PCB congeners (Reichrtova et al. 1999). In an *in vitro* study, treatment with PCB-118, but not PCB-153, led to a shift in the differentiation of CD4^+^ T lymphocytes toward a T-helper 2–dominated response, which is consistent with allergic disease (Gaspar-Ramirez et al. 2012).

We are not aware of previous studies specifically designed to examine developmental HCB exposures and the risk of offspring asthma. HCB was not associated with wheezing or asthma in a cross-sectional study of 124 Japanese adults (Miyake et al. 2011), but HCB has been found to induce airway hyperreactivity in tissue from HCB-exposed Brown Norway rats (Michielsen C et al. 2002; Michielsen CP et al. 2001). HCB has also been reported to show some agonist activity with the AhR (van Birgelen 1998), which suggests that HCB might affect the immune system and asthma through AhR-mediated mechanisms.

In contrast to two previous studies (Karmaus et al. 2001; Sunyer et al. 2005), we did not find any associations between maternal concentrations of *p,p*´-DDE and offspring asthma. It is worth noting that the children in the previous studies were younger (4–9 years of age) than our study population, and their findings may therefore at least partly reflect associations with wheezing symptoms that often resolve later in childhood (Bel 2004; Henderson et al. 2008; Taussig et al. 2003).

Breastfeeding may play an important role on the development of the infant’s immune system (Belderbos et al. 2012). On the other hand, breastfeeding is also a major source of exposure to PCBs and other lipophilic pollutants during the first year of life, with accumulation that is proportional to maternal concentrations (Wang et al. 2004) and the duration of breastfeeding (Patandin et al. 1997). In a study of prenatal and lactational exposures to PCBs in a relatively highly exposed fishing community (Grandjean et al. 2010), duration of breastfeeding was strongly correlated with offspring IgE concentrations at 7 years of age, whereas only a nonsignificant positive association was observed for prenatal PCB exposures. Although the focus of that study was on allergic sensitization and not asthma, the results support the potential importance of lactational POP exposures to immunoregulation. Absence of information on breastfeeding in our study is therefore a major limitation. As a result, we therefore cannot determine to which degree the associations observed in our study may be related to *in utero* exposure, lactational exposures, or a combination of both. Although speculative, previous surveys suggest that most children in Denmark were exclusively breastfed during the study’s time period (Vestergaard et al. 1999), which may possibly reduce potential confounding by breastfeeding.

The long prospective follow-up period and the high inclusion of 90% (872/965) of the offspring of women originally enrolled in the cohort are major strengths of our study. In contrast to previous studies that have mostly relied on self-reported asthma, our outcome measure was based on objective register data, where misclassification of ICD-10 asthma diagnoses has been shown to be low (Østergaard Jensen et al. 2010). A possible limitation of the definition of asthma based on medication data is that medicine may have been prescribed to clarify a diagnosis, and this method may therefore overestimate the true prevalence of disease; however, we used a definition based on medication data that has been validated against medical records with a sensitivity of 63% and specificity of 86% compared with a doctor diagnosis of asthma (Moth et al. 2007). Furthermore, we did not consider prescriptions before 6 years of age, which we believe is a strength because many children are prescribed asthma medications in the first years of life, although they do not necessarily develop clinical asthma (Ingvardsen et al. 2000). In most analyses with PCBs and HCB, we observed positive associations with the outcomes. The analyses based on the hospital diagnoses and the self-reported diagnoses of asthma did not always reach statistical significance, possibly due to a small number of cases and fewer subjects in the analyses, respectively. The significant associations observed between PCB-118 and HCB and self-reported current use of asthma medication may suggest that the associations are driven by persistent cases of asthma that require continuous medical treatment at 20 years of age.

As in any other observational study, we cannot exclude the possibility that the observed associations were influenced by residual or unmeasured confounding, including breastfeeding. The POPs included in our study were highly correlated, and consequently we could not distinguish the contributions of individual compounds to the observed associations. The potential confounders that we included as adjustment factors in our regression models seemed to influence our results to a minimal degree, suggesting limited residual confounding.

In conclusion, this study provides epidemiologic evidence that maternal concentrations of dioxin-like PCBs and HCB may be associated with the offspring’s risk of developing asthma that persists into adulthood. However, we cannot exclude the possibility that these associations may be mediated through postnatal exposures to POPs. Our results provide support for the hypothesis that early-life exposure to POPs may have immunoregulatory effects. Although the concentrations of these particular environmental contaminants have generally decreased since the late 1980s, industrial reduction in production and usage of one particular class of contaminants has typically been matched with a corresponding rise in the production and usage of other related industrial chemicals (Fangstrom et al. 2008; Haug et al. 2009) that have been less extensively studied with respect to potential health effects. In addition, evidence regarding the potential health effects of POPs will continue to be relevant to populations with high risks of occupational exposures (Schettgen et al. 2012) and to communities with high consumption of fish and other marine species (Birgisdottir et al. 2012).

## References

[r1] Asher MI, Keil U, Anderson HR, Beasley R, Crane J, Martinez F (1995). International Study of Asthma and Allergies in Childhood (ISAAC): rationale and methods.. Eur Respir J.

[r2] Bel EH (2004). Clinical phenotypes of asthma.. Curr Opin Pulm Med.

[r3] Belderbos ME, Houben ML, van Bleek GM, Schuijff L, van Uden NO, Bloemen-Carlier EM (2012). Breastfeeding modulates neonatal innate immune responses: a prospective birth cohort study.. Pediatr Allergy Immunol.

[r4] Birgisdottir BE, Brantsaeter AL, Kvalem HE, Knutsen HK, Haugen M, Alexander J (2012). Fish liver and seagull eggs, vitamin D-rich foods with a shadow: results from the Norwegian Fish and Game Study.. Mol Nutr Food Res.

[r5] Bjermo H, Darnerud PO, Lignell S, Pearson M, Rantakokko P, Nalsen C (2013). Fish intake and breastfeeding time are associated with serum concentrations of organochlorines in a Swedish population.. Environ Int.

[r6] Chiba T Chihara J Furue M 2012 Role of the Arylhydrocarbon Receptor (AhR) in the Pathology of Asthma and COPD. J Allergy (Cairo) 2012 372384; 10.1155/2012/372384 PMC3303582 22500183

[r7] Covaci A, Jorens P, Jacquemyn Y, Schepens P (2002). Distribution of PCBs and organochlorine pesticides in umbilical cord and maternal serum.. Sci Total Environ.

[r8] Dallaire F Dewailly É Vezina C Muckle G Weber JP Bruneau S 2006 Effect of prenatal exposure to polychlorinated biphenyls on incidence of acute respiratory infections in preschool Inuit children. Environ Health Perspect 114 1301 1305; 10.1289/ehp.8683 16882544 PMC1552004

[r9] Dyke PH, Foan C, Fiedler H (2003). PCB and PAH releases from power stations and waste incineration processes in the UK.. Chemosphere.

[r10] Ezendam J, Vos JG, Pieters R (2005). Research articles mechanisms of hexachlorobenzene-induced adverse immune effects in Brown Norway rats.. J Immunotoxicol.

[r11] Fangstrom B, Athanassiadis I, Odsjo T, Noren K, Bergman A (2008). Temporal trends of polybrominated diphenyl ethers and hexabromocyclododecane in milk from Stockholm mothers, 1980–2004.. Mol Nutr Food Res.

[r12] Gaspar-Ramirez O, Perez-Vazquez FJ, Pruneda-Alvarez LG, Orta-Garcia ST, Gonzalez-Amaro R, Perez-Maldonado IN (2012). Effect of polychlorinated biphenyls 118 and 153 on Th1/Th2 cells differentiation.. Immunopharmacol Immunotoxicol.

[r13] Glynn A Thuvander A Aune M Johannisson A Darnerud PO Ronquist G 2008 Immune cell counts and risks of respiratory infections among infants exposed pre- and postnatally to organochlorine compounds: a prospective study. Environ Health 7 62; 10.1186/1476-069X-7-62 19055819 PMC2637846

[r14] Grandjean P Poulsen LK Heilmann C Steuerwald U Weihe P 2010 Allergy and sensitization during childhood associated with prenatal and lactational exposure to marine pollutants. Environ Health Perspect 118 1429 1433; 10.1289/ehp.1002289 20562055 PMC2957924

[r15] Halldorsson TI, Meltzer HM, Thorsdottir I, Knudsen V, Olsen SF (2007). Is high consumption of fatty fish during pregnancy a risk factor for fetal growth retardation? A study of 44,824 Danish pregnant women.. Am J Epidemiol.

[r16] Halldorsson TI, Thorsdottir I, Meltzer HM, Nielsen F, Olsen SF (2008). Linking exposure to polychlorinated biphenyls with fatty fish consumption and reduced fetal growth among Danish pregnant women: a cause for concern?. Am J Epidemiol.

[r17] Haug LS, Thomsen C, Becher G (2009). Time trends and the influence of age and gender on serum concentrations of perfluorinated compounds in archived human samples.. Environ Sci Technol.

[r18] Heilmann C Grandjean P Weihe P Nielsen F Budtz-Jørgensen E 2006 Reduced antibody responses to vaccinations in children exposed to polychlorinated biphenyls. PLoS Med 3 e311; 10.1371/journal.pmed.0030311 16942395 PMC1551916

[r19] Henderson J, Granell R, Heron J, Sherriff A, Simpson A, Woodcock A (2008). Associations of wheezing phenotypes in the first 6 years of life with atopy, lung function and airway responsiveness in mid-childhood.. Thorax.

[r20] Ingvardsen BK Kampmann JM Laursen LC Johansen HL 2000 Utilization of anti-asthmatic drugs among Danish children in 1998 [in Danish] Ugeskr Laeger 162 6062 6065 11107942

[r21] Jusko TA, De Roos AJ, Schwartz SM, Lawrence BP, Palkovicova L, Nemessanyi T (2011). Maternal and early postnatal polychlorinated biphenyl exposure in relation to total serum immunoglobulin concentrations in 6-month-old infants.. J Immunotoxicol.

[r22] Karmaus W, Kuehr J, Kruse H (2001). Infections and atopic disorders in childhood and organochlorine exposure.. Arch Environ Health.

[r23] Longnecker MP Wolff MS Gladen BC Brock JW Grandjean P Jacobson JL 2003 Comparison of polychlorinated biphenyl levels across studies of human neurodevelopment. Environ Health Perspect 111 65 70; 10.1289/ehp.546 12515680 PMC1241307

[r24] Lundqvist C, Zuurbier M, Leijs M, Johansson C, Ceccatelli S, Saunders M (2006). The effects of PCBs and dioxins on child health.. Acta Paediatr Suppl.

[r25] Michielsen C, Zeamari S, Leusink-Muis A, Vos J, Bloksma N (2002). The environmental pollutant hexachlorobenzene causes eosinophilic and granulomatous inflammation and in vitro airways hyperreactivity in the Brown Norway rat.. Arch Toxicol.

[r26] Michielsen CC, van Loveren H, Vos JG (1999). The role of the immune system in hexachlorobenzene-induced toxicity.. Environ Health Perspect.

[r27] Michielsen CP, Leusink-Muis A, Vos JG, Bloksma N (2001). Hexachlorobenzene-induced eosinophilic and granulomatous lung inflammation is associated with *in vivo* airways hyperresponsiveness in the Brown Norway rat.. Toxicol Appl Pharmacol.

[r28] Miyake Y, Tanaka K, Masuzaki Y, Sato N, Ikeda Y, Chisaki Y (2011). Organochlorine concentrations in breast milk and prevalence of allergic disorders in Japanese women.. Chemosphere.

[r29] Mohammed A, Eklund A, Ostlund-Lindqvist AM, Slanina P (1990). Distribution of toxaphene, DDT, and PCB among lipoprotein fractions in rat and human plasma.. Arch Toxicol.

[r30] Moth G, Vedsted P, Schiotz P (2007). Identification of asthmatic children using prescription data and diagnosis.. Eur J Clin Pharmacol.

[r31] National Board of Health, Denmark. (1977). International Classification of Diseases, 8th Revision (ICD-8) [in Danish].

[r32] National Board of Health, Denmark. (1994). International Classification of Diseases, 10th Revision [in Danish].

[r33] Olsen SF, Hansen HS, Secher NJ, Jensen B, Sandstrom B (1995). Gestation length and birth weight in relation to intake of marine n-3 fatty acids.. Br J Nutr.

[r34] Østergaard Jensen A, Nielsen GL, Ehrenstein V (2010). Validity of asthma diagnoses in the Danish National Registry of Patients, including an assessment of impact of misclassification on risk estimates in an actual dataset.. Clin Epidemiol.

[r35] Park JS, Bergman A, Linderholm L, Athanasiadou M, Kocan A, Petrik J (2008). Placental transfer of polychlorinated biphenyls, their hydroxylated metabolites and pentachlorophenol in pregnant women from eastern Slovakia.. Chemosphere.

[r36] Patandin S, Weisglas-Kuperus N, de Ridder MA, Koopman-Esseboom C, van Staveren WA, van der Paauw CG (1997). Plasma polychlorinated biphenyl levels in Dutch preschool children either breast-fed or formula-fed during infancy.. Am J Public Health.

[r37] Peat JK, Salome CM, Toelle BG, Bauman A, Woolcock AJ (1992). Reliability of a respiratory history questionnaire and effect of mode of administration on classification of asthma in children.. Chest.

[r38] Peat JK, Toelle BG, Marks GB, Mellis CM (2001). Continuing the debate about measuring asthma in population studies.. Thorax.

[r39] Rantakokko P, Kiviranta H, Rylander L, Rignell-Hydbom A, Vartiainen T (2009). A simple and fast liquid-liquid extraction method for the determination of 2,2’,4,4’,5,5’-hexachlorobiphenyl (CB-153) and 1,1-dichloro-2,2-bis(*p*-chlorophenyl)-ethylene (*p,p*´-DDE) from human serum for epidemiological studies on type 2 diabetes.. J Chromatogr A.

[r40] Reichrtova E, Ciznar P, Prachar V, Palkovicova L, Veningerova M (1999). Cord serum immunoglobulin E related to the environmental contamination of human placentas with organochlorine compounds.. Environ Health Perspect.

[r41] Schettgen T, Gube M, Esser A, Alt A, Kraus T (2012). Plasma polychlorinated biphenyls (PCB) levels of workers in a transformer recycling company, their family members, and employees of surrounding companies.. J Toxicol Environ Health A.

[r42] Stolevik SB, Nygaard UC, Namork E, Haugen M, Kvalem HE, Meltzer HM (2011). Prenatal exposure to polychlorinated biphenyls and dioxins is associated with increased risk of wheeze and infections in infants.. Food Chem Toxicol.

[r43] Sunyer J Torrent M Munoz-Ortiz L Ribas-Fito N Carrizo D Grimalt J 2005 Prenatal dichlorodiphenyldichloroethylene (DDE) and asthma in children. Environ Health Perspect 113 1787 1790; 10.1289/ehp.8127 16330365 PMC1314922

[r44] Taussig LM, Wright AL, Holberg CJ, Halonen M, Morgan WJ, Martinez FD (2003). Tucson Children’s Respiratory Study: 1980 to present.. J Allergy Clin Immunol.

[r45] Thompson MR, Boekelheide K (2013). Multiple environmental chemical exposures to lead, mercury and polychlorinated biphenyls among childbearing-aged women (NHANES 1999–2004): body burden and risk factors.. Environ Res.

[r46] van Birgelen AP (1998). Hexachlorobenzene as a possible major contributor to the dioxin activity of human milk.. Environ Health Perspect.

[r47] Vestergaard M, Obel C, Henriksen TB, Sorensen HT, Skajaa E, Ostergaard J (1999). Duration of breastfeeding and developmental milestones during the latter half of infancy.. Acta Paediatr.

[r48] Wang SL, Lin CY, Guo YL, Lin LY, Chou WL, Chang LW (2004). Infant exposure to polychlorinated dibenzo-*p*-dioxins, dibenzofurans and biphenyls (PCDD/Fs, PCBs)–correlation between prenatal and postnatal exposure.. Chemosphere.

[r49] Weisglas-Kuperus N, Patandin S, Berbers GA, Sas TC, Mulder PG, Sauer PJ (2000). Immunologic effects of background exposure to polychlorinated biphenyls and dioxins in Dutch preschool children.. Environ Health Perspect.

[r50] Weisglas-Kuperus N, Vreugdenhil HJ, Mulder PG (2004). Immunological effects of environmental exposure to polychlorinated biphenyls and dioxins in Dutch school children.. Toxicol Lett.

